# Case report: Guillain-Barré syndrome following acute ischemic brainstem stroke

**DOI:** 10.3389/fimmu.2024.1403561

**Published:** 2024-11-27

**Authors:** Da Xu, Cheng Yu, Xue-Jun Deng, Man Li

**Affiliations:** Department of Neurology, Union Hospital, Tongji Medical College, Huazhong University of Science and Technology, Wuhan, Hubei, China

**Keywords:** Guillain-Barré syndrome, acute ischemic stroke, intravenous immunoglobulin, diagnosis, case report

## Abstract

Guillain–Barré syndrome (GBS) is a heterogeneous disease, and it usually develops after an antecedent infection, while rare cases develop following a central nervous system disease. Given the indeterminacy of prodromal infection symptoms and the overlap of symptoms with other neurological disorders, a rigorous clinical and neurological examination in conjunction with history is necessary for early diagnosis and treatment of GBS. Here we present a rare case of GBS following acute ischemic stroke which was different from previous reports such as GBS following hemorrhagic stroke or head trauma. Moreover, intravenous immunoglobulin was effective in this patient after 6 months of follow-up despite the potential risk of thrombotic events.

## Introduction

Guillain–Barré syndrome (GBS) is an acute inflammatory demyelinating neuropathy typically following a mild respiratory or gastrointestinal viral infection (1). Intravenous immunoglobulin (IVIg) is generally recommended as the first-line therapy for GBS, although it may cause thromboembolism in patients with vascular risks (2, 3). It was reported that GBS could develop following an acute central nervous system disease such as head trauma, neurosurgery, and cerebral hemorrhage (4). Generally, stroke could be easily distinguished from GBS in many aspects (**Table 1**). Here we report a patient with GBS following acute ischemic brainstem stroke which was previously rarely reported, and IVIg showed profound therapeutic effects (5).

**Table 1:** Clinical differences between stroke and GBS.

Disease	Stroke	GBS
Underlying disease	Hypertension, diabetes mellitus, hyperlipemia, smoke, alcohol, etc.	respiratory or gastrointestinal viral infection
Clinical manifestations	Especially on one side of the body, sudden numbness or weakness, cognitive impairment, vision problems, headaches, dizziness, etc.	Usually symmetrical strength or sensory impairment
Neurological signs	Tendon hyperreflexia, pathological signs (+)	Tendon reflex drops or disappears, pathological signs (-)
Pathogenesis	Cerebral vascular disease	inflammatory demyelinating neuropathy
Auxiliary examination	EMG (-), head MRI (+), CSF (-)	EMG (+), head MRI (-), CSF (+)

EMG, electromyography; MRI, magnetic resonance imaging; CSF, cerebrospinal fluid.

## Case presentation

**Figure 1:**
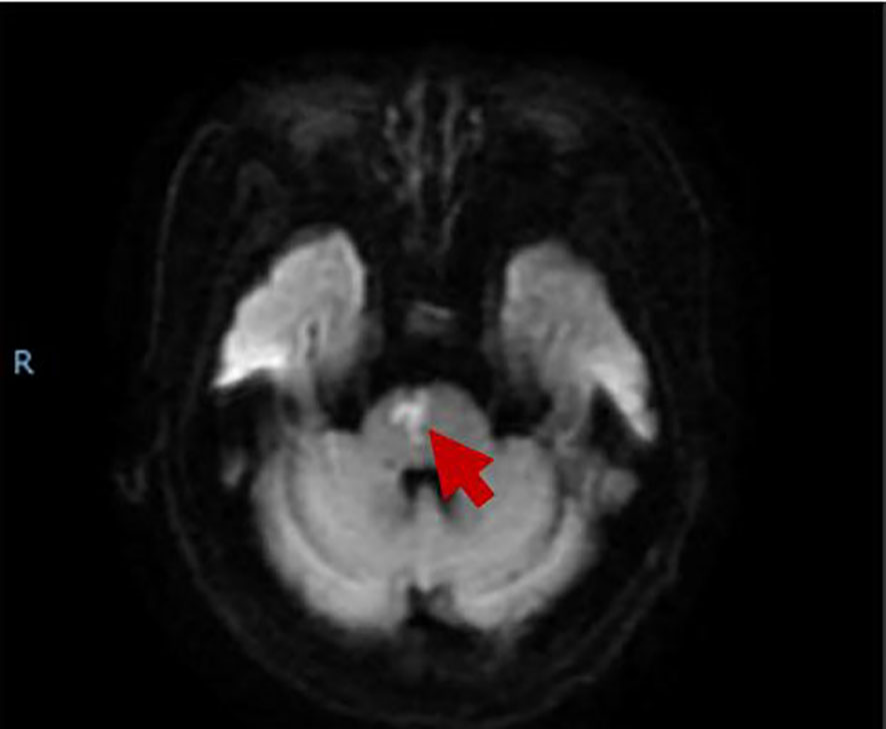
The MRI showed acute brainstem infarction.

A 74-year-old woman was hospitalized for sudden onset of left limb weakness and numbness with walking instability. There were no risk factors for vascular diseases such as hypertension, diabetes, hyperlipidemia, smoking, and drinking. Neurological examination showed that the patient had dysarthria, grade 4 muscle strength in the proximal and grade 2 muscle strength in the distal muscles of the left limbs, normal muscle tonus and hyperactive tendon reflexes in the left limbs, and positive Babinski sign on the left. Her blood test showed an increase in D-dimer level (0.81, normal range: <0.5), while the rest showed no significant abnormalities. Magnetic resonance imaging (MRI) indicated acute paramedian pons infarction (**Figure 1**), bilateral multiple lacunar cerebral infarctions, cerebral atherosclerosis, mild stenosis of bilateral vertebral artery V1 and right vertebral artery V4. No obvious abnormality was found in Doppler echocardiography and rhythm Holter. After a week of treatment for stroke including antiplatelet and lipid-lowering therapy, butyphthalide, and edaravone (drugs which are used in the treatment of acute ischemic stroke in China), the patient's symptoms improved, the muscle strength of the distal left upper limb returned to grade 4, the speech improved, while the rest of the physical examination was the same as before, and she was discharged home.

**Figure 2:**
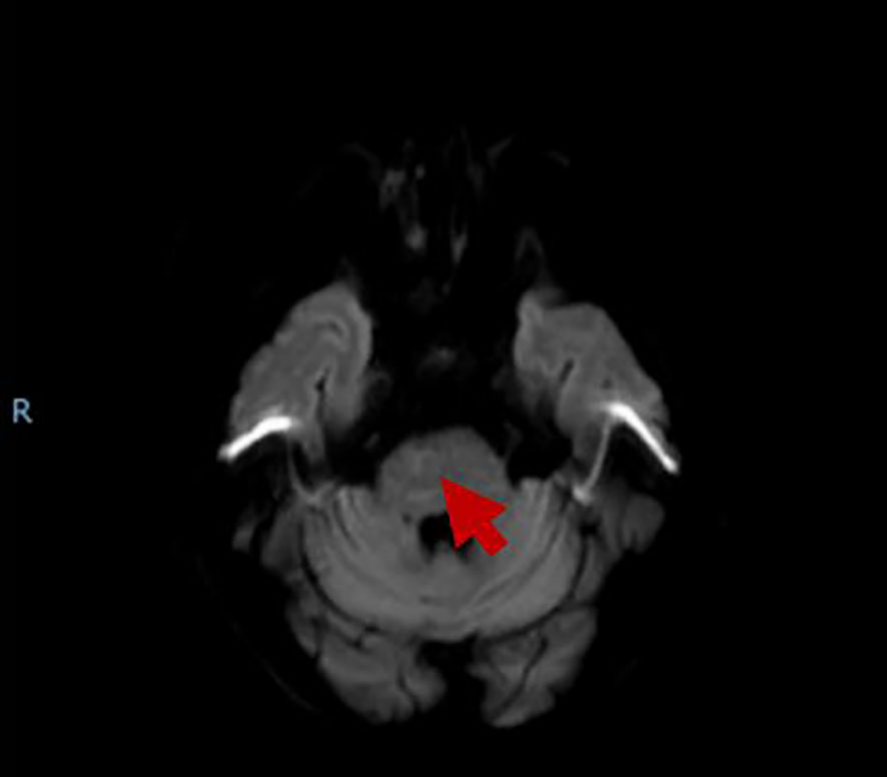
The MRI showed that brainstem infarction was subacute to chronic, and the restricted diffusion significantly reduced.

AT 3 days after discharge, the patient was hospitalized again, complaining of gradual weakness in the four limbs without severe infection history. Neurological examination showed that the patient had grade 3 of muscle strength in both upper limbs, grade 2 of muscle strength in both lower limbs, normal tendon reflexes, and negative Babinski's sign, Oppenheim's sign, Gordon's sign, and Chaddock's sign in both lower limbs. We were worried about the patient's relapse of cerebral infarction or hemorrhagic transformation although urgent head CT examination showed no bleeding or new infarction. Blood test showed a slight decrease in D-dimer level to 0.66, with no obvious abnormality in the rest. Head MRI showed that the ischemic infarction was in the subacute to chronic state, and the degree of restricted diffusion was significantly reduced (**Figure 2**). The symptoms of the patient treated as cerebral infarction did not improve. The results of lumbar puncture showed that the white blood cell count of cerebrospinal fluid (CSF) was 1*10^6^/L (normal range: 0–8*10^6^/L ), protein was 0.55 g/L (normal range: 0.15–0.45 ), glucose was 3.96 mmol/L ( normal range: 2.2–3.9 ), CSF-IgA was 9.01 mg/L (normal range: 0–7.00), and trace albumin was 503.0 mg/L (normal range: 139.0–246.0). No positive result was found in CSF virus tests including cytomegalovirus IgM, coxsackievirus B3 IgM, coxsackievirus B5 IgM, and enterovirus RNA. Anti-GD2 ganglioside antibody was detected (**Table 1**, appendixes). Electromyography showed that the motor conduction velocity of left and right peroneal nerves, left tibial nerve, and left ulnar nerve was reduced; the sensory conduction velocity of the left sural nerve was completely reduced. The F wave persistence of the left tibial nerve was decreased (37.5%), and the latency was prolonged. F wave was not recorded for the left ulnar nerve. H reflexes were not recorded in both lower limbs. Therefore, the patient was diagnosed as having acute GBS. After treatment with IVIg of 0.4 g/kg for 5 days, the patient's symptoms improved slightly. At follow-up after 6 months from discharge, the patient's symptoms significantly improved, with muscle strength of grade 5 in the four limbs.

## Discussion

Reports of GBS following acute ischemic brainstem stroke are rare. Nevertheless, there were several reports showing hyperacute GBS mimicking stroke or brainstem infarction mimicking GBS syndrome (6–8). In this patient with ischemic stroke, at the time the symptoms got worse, we firstly considered relapse of stroke or hemorrhagic transformation. GBS was considered after ineffective treatment for stroke, which should be noted in the future work. As a delayed or even wrong diagnosis of GBS may cause disability or even death when the respiratory function was depressed, it is important to ensure early diagnosis and timely treatment.

The mechanisms underlying GBS following stroke remained unknown, and immunoreaction was supposed to play a role. Damage-associated molecular patterns and cytokines expressed in the early stage of ischemic stroke can enter the systemic circulation through the damaged BBB or CSF drainage system (including venous and lymphatic outflow) (9). This early activation of the immune system can easily lead to systemic immunosuppression (10). Ischemia-damaged brain has a strong influence on the peripheral immune system by regulating the development and steady state of spleen and bone marrow immune cells, which creates conditions for patients to be infected and eventually leads to the occurrence of GBS (11). Besides this, antigen-dependent autoimmunity develops after stroke (12). For example, B lymphocyte reaction and the production of CNS antibody may increase with time, and about half of stroke survivors showed intrathecal antibody synthesis after the first week (13). Without more evidence, we could only speculate that immune disorder may be the reason of IgM anti-GD2 production. It was reported that IVIg might be associated with strokes (2, 3). Rarely do patients develop stroke after IVIg, perhaps due to hyperviscosity, thromboemboli, vasculitis, or cerebral vasospasm (2, 3). In some reports on GBS and cerebral hemorrhage, majority of the patients got partial or complete recovery from IVIg treatment (4). Moreover, the rate of cerebral infarction following IVIg is higher than hemorrhage (14). In this patient, IVIg did not worsen the symptoms of stroke; conversely, it improved the symptoms.

## Conclusions

Although ischemic stroke was concomitant with GBS or GBS developed secondary to ischemic stroke remained unknown, it is imperative to check the neurological function carefully to achieve a correct diagnosis, earlier treatment, and better prognosis.

## Data Availability

The original contributions presented in the study are included in the article/supplementary material. Further inquiries can be directed to the corresponding author.
